# The GSK3 kinase inhibitor lithium produces unexpected hyperphosphorylation of β-catenin, a GSK3 substrate, in human glioblastoma cells

**DOI:** 10.1242/bio.030874

**Published:** 2017-12-06

**Authors:** Ata ur Rahman Mohammed Abdul, Bhagya De Silva, Ronald K. Gary

**Affiliations:** Department of Chemistry and Biochemistry, University of Nevada Las Vegas, Las Vegas, NV 89154, USA

**Keywords:** Glycogen synthase kinase, β-Catenin, Phosphorylation, Wnt signaling, Axin, Capillary electrophoresis

## Abstract

Lithium salt is a classic glycogen synthase kinase 3 (GSK3) inhibitor. Beryllium is a structurally related inhibitor that is more potent but relatively uncharacterized. This study examined the effects of these inhibitors on the phosphorylation of endogenous GSK3 substrates. In NIH-3T3 cells, both salts caused a decrease in phosphorylated glycogen synthase, as expected. GSK3 inhibitors produce enhanced phosphorylation of Ser9 of GSK3β via a positive feedback mechanism, and both salts elicited this enhancement. Another GSK3 substrate is β-catenin, which has a central role in Wnt signaling. In A172 human glioblastoma cells, lithium treatment caused a surprising increase in phospho-Ser33/Ser37-β-catenin, which was quantified using an antibody-coupled capillary electrophoresis method. The β-catenin hyperphosphorylation was unaffected by p53 RNAi knockdown, indicating that p53 is not involved in the mechanism of this response. Lithium caused a decrease in the abundance of axin, a component of the β-catenin destruction complex that has a role in coordinating β-catenin ubiquitination and protein turnover. The axin and phospho-β-catenin results were reproduced in U251 and U87MG glioblastoma cell lines. These observations run contrary to the conventional view of the canonical Wnt signaling pathway, in which a GSK3 inhibitor would be expected to decrease, not increase, phospho-β-catenin levels.

This article has an associated First Person interview with the first author of the paper.

## Abstract (precis)

**Summary:** GSK3 inhibitors have potential use against Alzheimer's disease and other conditions. In this study, a classic inhibitor produced unexpected molecular effects on key components of the Wnt signaling pathway.

## INTRODUCTION

Glycogen synthase kinase 3 (GSK3) has multiple functions in cell growth and differentiation (Frame and Cohen, 2001; Doble and Woodgett, 2003; McNeill and Woodgett, 2010; Beurel et al., 2015). It has a central role in insulin signaling and in the canonical Wnt signaling pathway. In mammals, GSK3 exists as two isoforms, alpha and beta, that are often co-expressed in various cell and tissue types. GSK3α and GSK3β appear to have generally overlapping biological roles and they have been shown to be functionally redundant in various contexts (Asuni et al., 2006; Doble et al., 2007), although only GSK3β is essential for viability in mouse knockout models (Hoeflich et al., 2000; Kaidanovich-Beilin et al., 2009). The kinase domains of the two isoforms share 98% amino acid sequence identity (Doble and Woodgett, 2003), so kinase inhibitors are unlikely to differentiate between GSK3α and GSK3β.

GSK3 is constitutively active, and hormones and other molecules that mediate signaling through GSK3-containing pathways typically trigger a reduction in GSK3 kinase activity. The insulin signal transduction pathway provides a clear example of this. GSK3 phosphorylates glycogen synthase (GS) at a cluster of residues (Ser641, Ser645, and Ser649) to maintain GS in a dormant state (Rylatt et al., 1980; Kaidanovich and Eldar-Finkelman, 2002). Insulin causes GSK3 to become phosphorylated at Ser21 of GSK3α and at the homologous position, Ser9, in GSK3β (Cross et al., 1995). These are inhibitory post-translational modifications that diminish the kinase activity of GSK3, so insulin signaling relieves GSK3-mediated suppression of GS activity and results in the synthesis of glycogen. This is the central mechanism by which insulin leads to carbohydrate storage during the ‘well-fed’ state of energy abundance. Drugs that inhibit GSK3 kinase activity mimic the action of insulin (Kaidanovich and Eldar-Finkelman, 2002; Patel et al., 2004). For example, lithium chloride, an archetypal GSK3 inhibitor, stimulates glucose transport and GS activity in adipocytes (Cheng et al., 1983a,b; Oreña et al., 2000).

In the canonical Wnt signaling pathway, GSK3 partners with APC, axin, and other proteins to form a β-catenin ‘destruction complex’ (Doble and Woodgett, 2003; Patel et al., 2004; McNeill and Woodgett, 2010). With the aid of APC and axin, which provide scaffolding functions, GSK3 phosphorylates β-catenin at Ser33, Ser37, and Thr41 (Doble and Woodgett, 2003; Valvezan et al., 2012; Stamos and Weis, 2013). The phospho-Ser33 and phospho-Ser37 pair provides the recognition site for the β-TrCP E3-ubiquitin ligase (Patel et al., 2004; Stamos and Weis, 2013). The ubiquitinated phospho-β-catenin undergoes degradation in the proteasome. This process results in continuous turnover of β-catenin, holding protein levels in check. When the Wnt ligand binds to its receptor, a chain of events is initiated that disrupts the function of the destruction complex and prevents the phosphorylation and destruction of β-catenin. The β-catenin protein accumulates and is transported to the nucleus, where it stimulates TCF/LEF transcription factors to cause upregulation of proliferation-associated targets such as c-myc, cyclin D1, and many others.

Modulators of GSK3 activity have potential as therapeutic agents. GSK3 inhibitors could be useful in the treatment of Type 2 diabetes. GSK3 inhibitors are also being considered for their potential to ameliorate the progression of Alzheimer's disease. Neurofibrillary tangles and amyloid plaques are histological hallmarks of this disease. The neurofibrillary tangles are composed of hyperphosphorylated tau protein, and GSK3 is thought to be the principal kinase responsible for tau phosphorylation. Therefore, GSK3 inhibitors could suppress tau phosphorylation in brain tissue and thereby decrease the prevalence of neurofibrillary tangles. With respect to Wnt signaling, the effects of GSK3 inhibitors could be problematic. Turnover of β-catenin protein requires the phosphorylation of β-catenin by GSK3. Inhibition of GSK3 should lead to hypophosphorylation of β-catenin, increased abundance, and nuclear accumulation, which would promote the transcription of growth promoting gene products. In the context of the conventional view of Wnt signaling, one would have to consider the possibility that GSK3 inhibitors could be carcinogenic.

Lithium salt is a classic GSK3 inhibitor (Stambolic et al., 1996; Hedgepeth et al., 1997; Ryves and Harwood, 2001; Jope, 2003; O'Brien and Klein, 2009). Synthetic organic GSK3 inhibitors with greater potency have been identified, but lithium remains the most widely utilized inhibitor in cell culture studies and in animal models. As a potential therapeutic, lithium has one great advantage compared to the newer synthetic compounds: lithium salt is FDA-approved for treatment of bipolar disorder, and there is a record of safety and efficacy that extends for many decades. There is currently an especially strong interest in lithium as a possible therapy for Alzheimer's disease. Many studies have reported that lithium produces beneficial cognitive effects in mouse models of Alzheimer's disease (King et al., 2014), including not only tau-specific models but also in transgenic mice that express a mutant form of the amyloid precursor protein (Nunes et al., 2015). Controlled clinical trials are showing encouraging indications that patients with Alzheimer's might benefit from lithium therapy (Matsunaga et al., 2015; Forlenza et al., 2016). Interestingly, despite the theoretical potential that lithium might mimic the cell growth-promoting aspects of Wnt signaling, epidemiological evidence does not support increased cancer risk in the population of bipolar patients who have been treated with lithium (Cohen et al., 1998; Martinsson et al., 2016; Huang et al., 2016).

GSK3 uses Mg^2+^ ion as a cofactor. Lithium and beryllium (atomic numbers 3 and 4) have an ionic radius smaller than that of the magnesium ion (atomic number 12). Like Li^+^, Be^2+^ inhibits GSK3 kinase activity, but beryllium is about 1000-fold more potent (Ryves et al., 2002; Mudireddy et al., 2014). GSK3 that has been immunoprecipitated from cells that were treated with Li^+^ or Be^2+^ has reduced kinase activity (Mudireddy et al., 2014). The present study set out to examine the phosphorylation status of endogenous GSK3 substrates in cells that had been treated with Be^2+^, using Li^+^ treatment for comparison and as a positive control. We found that both metal salts caused a decrease in the phosphorylation of cellular GS, as expected. According to the standard model of Wnt signaling, GSK3 inhibitors should cause a decrease in the phosphorylation of cellular β-catenin as well, but that is not what we observed. In A172 human glioblastoma cells, Li^+^ treatment caused a surprising increase in phospho-Ser33/Ser37-β-catenin, which was the opposite of the anticipated effect. This observation was reproduced in other glioma cell lines, and to some extent in non-glial cell types. These results suggest that the simple relationship between lithium, GSK3 activity, and β-catenin phosphorylation that is portrayed in most models of canonical Wnt signaling may not be consistently applicable.

## RESULTS

### Li^+^ and Be^2+^ cause decreased phosphorylation of glycogen synthase at Ser641/Ser645 in NIH-3T3 cells

This study aimed to investigate the effects of lithium and beryllium salts on the phosphorylation of endogenous GSK3 substrates. In order to assess more normal physiological conditions, untransfected cells were used instead of cells overexpressing recombinant substrates. In NIH-3T3 mouse embryo fibroblast cells, 20 mM Li^+^ caused a decrease in phosphorylation of endogenous GS at a site that is known to be a substrate for GSK3, whereas 20 mM K^+^ had no effect (Fig. S1). Be^2+^ at 30 and 100 µM produced a similar decrease, whereas 100 µM Ca^2+^ had no effect. Li^+^ and Be^2+^ did not change the levels of total GS, total GSK3α, or total GSK3β.

### Li^+^ and Be^2+^ cause increased phosphorylation of GSK3β at Ser9 in NIH-3T3 cells

GSK3 inhibitors elicit an increase in the phosphorylation of GSK3β at the Ser9 position. This is because GSK3 inhibitory effects are amplified via a positive feedback loop involving a GSK3-dependent phosphatase activity (Jope, 2003). Phosphorylation at Ser9 depresses kinase activity. The PP1 phosphatase that dephosphorylates Ser9 requires GSK3β kinase activity to maintain its full activity (Zhang et al., 2003). Therefore, kinase inhibition leads to phosphatase inhibition, which promotes hyperphosphorylation of GSK3β at Ser9. This modification at Ser9 further suppresses kinase activity, amplifying the original effect. Thus, an increase in Ser9 phosphorylation is a hallmark of pharmacological GSK3β inhibition, and such a response was observed for both Li^+^ and Be^2+^ in NIH-3T3 cells (Fig. S1). However, previous studies using A172 cells showed that, in that cell type, increased Ser9-GSK3β phosphorylation can be elicited by 20 mM Li^+^ but not by 30 or 100 µM Be^2+^ (Mudireddy et al., 2014). Considering the divergent observations with respect to Be^2+^, we conducted additional experiments. The ability of BeSO_4_ to increase phospho-Ser9 antigen abundance in NIH-3T3 cells was confirmed by additional western blots (Fig. S2), in which the normalization was based on the total protein concentration of the cell lysates, and by flow cytometry analysis (Fig. S3), in which the normalization was on a per cell basis. In the flow cytometry assay, the mean fluorescence increased by 113%, 24%, and 143% for 20 mM Li^+^, 30 µM Be^2+^, and 100 µM Be^2+^, respectively, compared to untreated control cells. Thus, the consequences of Be^2+^ treatment with respect to this phosphorylation event appear to be cell type-dependent.

### Surprisingly, Li^+^ causes increased phosphorylation of β-catenin at Ser33 in A172 cells

The results with phospho-GS and phospho-Ser9-GSK3β in NIH-3T3 cells showed that Li^+^ and Be^2+^ act as GSK3 inhibitors when added to cell culture media. We next sought to examine the effects of these salts on β-catenin. In western blots, β-catenin bands were faint with NIH-3T3 cells. Therefore, A172 human glioblastoma cells were selected for study. These cells express the protein well, and the responsiveness of this cell type to the cytostatic effects of Li^+^ and Be^2+^ has been characterized (Gorjala and Gary, 2010; Mudireddy et al., 2014; Gorjala et al., 2016).

**Fig. 1.**
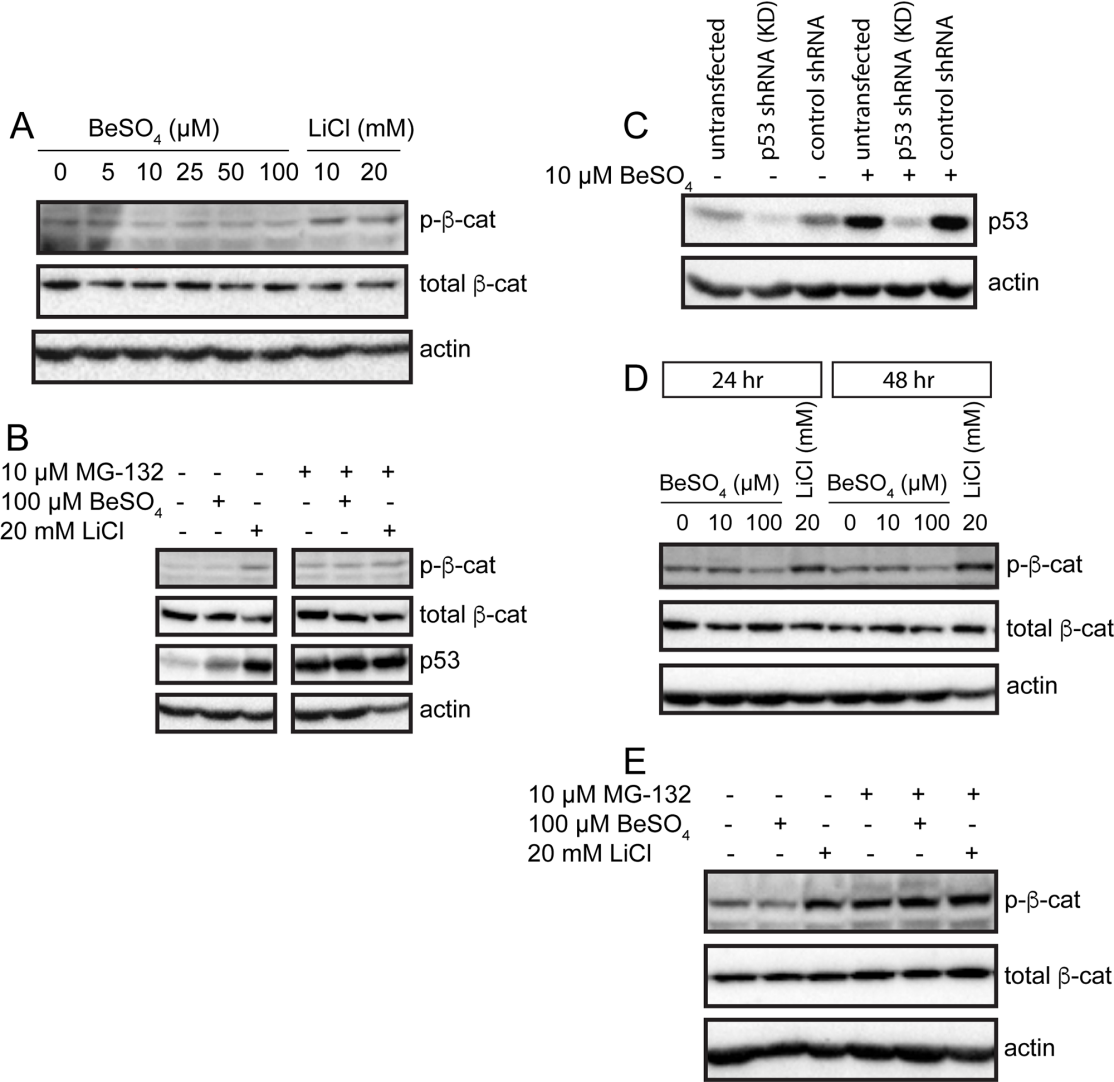
**Lithium causes increased phosphorylation of β-catenin.** Western blots show p-β-catenin (phospho-Ser33-β-catenin), total β-catenin, p53, and actin. (A) A172 cells were grown in the presence of the indicated concentrations of BeSO_4_ or LiCl for 24 h. (B) A172 cells were grown in 100 µM BeSO_4_ or 20 mM LiCl for 24 h, with or without the proteasome inhibitor MG-132 (10 µM) for 6 h. (C) Untransfected A172 cells, an A172 cell line transfected with p53 knock-down (KD) shRNA, or a control cell line transfected with an untargeted shRNA sequence were grown in the presence or absence of 10 µM BeSO_4_ for 24 h. (D) The p53 KD cells were grown in 10 µM BeSO_4_, 100 µM BeSO_4_, or 20 mM LiCl for 24 or 48 h. (E) The p53 KD cells were grown in 100 µM BeSO_4_ or 20 mM LiCl for 24 h, with or without the proteasome inhibitor MG-132 (10 µM) for 6 h.

Treatment of A172 cells with 10 mM and 20 mM LiCl caused Ser33 phosphorylation of β-catenin to increase (Fig. 1A). This result was unexpected for two reasons. First, a kinase inhibitor should decrease phosphorylation at GSK3 target sites, not increase it. Second, phosphorylation at this site provides a signal for ubiquitination and subsequent degradation by the proteasome, so proteolytic degradation should hinder accumulation of this species. To evaluate the role of proteasomal degradation in phospho-β-catenin turnover, the cells were treated with MG-132, a proteasome inhibitor (Fig. 1B). The expression of p53 was examined as a positive control; lithium and beryllium upregulate p53 (Lehnert et al., 2001; Gorjala and Gary, 2010; Mudireddy et al., 2014; Gorjala et al., 2016), and MG-132 prevents p53 degradation (Yang et al., 2005; Gorjala et al., 2016). Li^+^ and Be^2+^ caused an increase in p53, confirming the efficacy of the metal ion treatments, and MG-132 produced maximal p53 accumulation, confirming the efficacy of the MG-132 treatment (Fig. 1B). Blocking degradation for 6 h with MG-132 caused phospho-β-catenin to accumulate in control cells and Be^2+^-treated cells (Fig. 1B). This demonstrates that proteasome-mediated degradation of phospho-β-catenin is active in this cell type. For Li^+^-treated cells, the addition of MG-132 did not change phospho-β-catenin levels. This could be an indication that lithium is interfering with ubiquitination of phospho-β-catenin.

The p53 transcription factor has been shown to regulate β-catenin expression in some cell types (Sadot et al., 2001; Levina et al., 2004; Cha et al., 2012). Moreover, in HEK-293 cells, transfection with p53 causes increased β-catenin phosphorylation at Ser33 and Ser37 (Levina et al., 2004). If A172 and HEK-293 cells respond to p53 similarly, these studies suggest a possible mechanism that could explain the lithium result. A172 cells possess wild-type p53, and lithium activates p53 in these cells (Fig. 1B). Subsequently, p53 could cause increased β-catenin phosphorylation, if the HEK-293 results are also applicable to A172 cells. We wished to test the hypothesis that the lithium-induced changes in phospho-β-catenin could be mediated through p53. Therefore, we used a p53 knockdown A172 cell line to remove p53 effects. By comparing the lithium response in the presence and absence of p53, the possible involvement of p53 in the mechanism of β-catenin hyperphosphorylation could be evaluated. The p53 knockdown greatly reduced levels of p53 protein (Fig. 1C). In these knockdown cells, lithium increased phospho-β-catenin (Fig. 1D), similar to its effect in normal A172 cells. Likewise, the MG-132 experiment produced similar results in the absence of p53 (Fig. 1E) as it had in the presence of p53 (Fig. 1B). These observations show that p53 is not involved in the mechanism of lithium-induced accumulation of phospho-β-catenin.

During MG-132 treatments, p53 and phospho-β-catenin accumulate in their non-ubiquitinated forms. This is because the intracellular pool of free ubiquitin is dynamic and becomes depleted when proteasome-mediated recycling is blocked by MG-132.

### A dose-response curve for Li^+^-induced changes in β-catenin phosphorylation

**Fig. 2.**
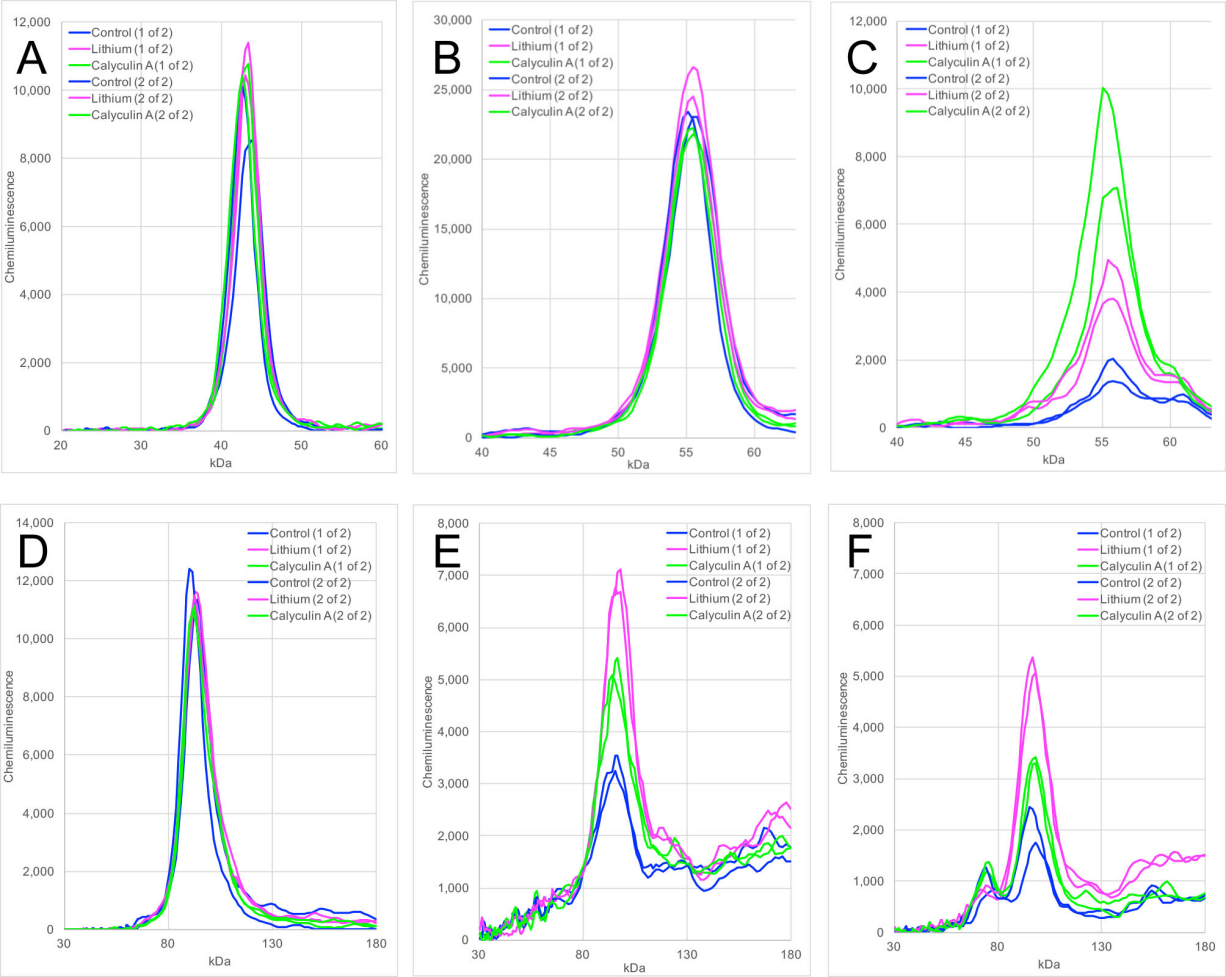
**Development of a capillary electrophoresis method for quantitative analysis of phosphoproteins.** A172 were treated with 20 mM LiCl for 24 h (pink curves) or 3 nM calyculin A for 1 h (green curves). Control cells were untreated (blue curves). There were two independent replicates per treatment. Cell lysates were subject to size separation by capillary electrophoresis, probed with six different primary antibodies, and secondary antibodies were used to generate a chemiluminescent signal. The electropherogram for each replicate is shown. (A) Peak for GAPDH. (B) Peak for total GSK3β. (C) Peak for phospho-Ser9-GSK3β. (D) Peak for total β-catenin. (E) Peak for phospho-Ser33/Ser37-β-catenin. (F) Peak for phospho-Ser33/Ser37/Thr41-β-catenin.

The lithium-induced changes in phospho-β-catenin were contrary to expectations, so an alternative approach was employed to corroborate the western blot results. A capillary electrophoresis method was developed that enabled improved quantitation and reproducibility. In western blotting, resolved proteins must be transferred to a nitrocellulose or PVDF membrane; in the capillary electrophoresis method, resolved proteins are immobilized *in situ* within the capillary via photochemical crosslinking. Thus, the variability associated with transfer efficiency is avoided with the capillary electrophoresis method. Automation of the blocking, staining, washing, and signal detection steps further enhances reproducibility. Proteins in cell lysates were separated on the basis of size, and a proportional relationship was observed between antigen abundance and the corresponding electropherogram peak area (Fig. S4). Next, the capillary electrophoresis method was validated as a means to quantitate phosphorylation levels in differentially treated cells (Fig. 2). As part of this process, cells were treated with calyculin A, a potent PP1/PP2A phosphatase inhibitor. The phosphatase inhibitor caused an increase in the phosphorylation of GSK3β and β-catenin, as expected, confirming the utility of the capillary electrophoresis method (Fig. 2C,E,F). Lithium, which is ostensibly a kinase inhibitor, also caused an increase in the phosphorylation of GSK3β and β-catenin (Fig. 2C,E,F). The first effect is readily explainable, via the positive feedback loop previously discussed, but the second effect is not. The treatments had little or no effect on the expression of the housekeeping gene GAPDH, total GSK3β, or total β-catenin (Fig. 2A,B,D). Interestingly, calyculin A treatment enhanced phosphorylation of GSK3β more than β-catenin, whereas lithium treatment enhanced phosphorylation of β-catenin more than GSK3β. During this initial characterization, two different p-β-catenin antibodies were tested: an affinity-purified rabbit polyclonal against human phospho-Ser33/Ser37-β-catenin (Fig. 2E), and an affinity-purified rabbit polyclonal against human phospho-Ser33/Ser37/Thr41-β-catenin (Fig. 2F). Both antibodies showed that lithium treatment increases phosphorylation at this cluster of GSK3 target sites. Because the two antibodies gave similar results, subsequent studies employed the phospho-Ser33/Ser37-β-catenin antibody alone.

**Fig. 3.**
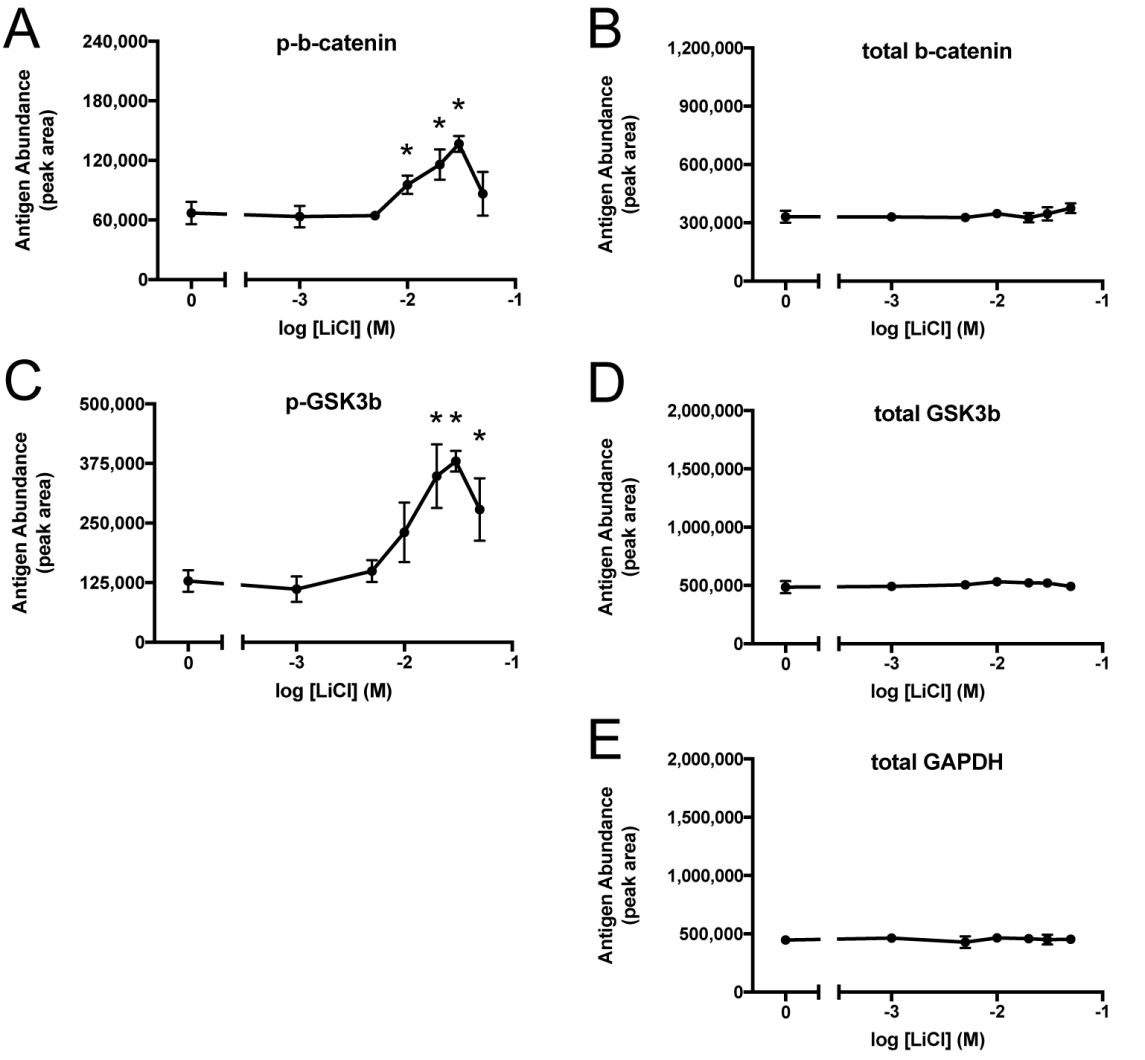
**Lithium dose-response curves for protein and phosphoprotein abundance using capillary electrophoresis.** A172 cells were treated with 0, 1, 5, 10, 20, 30, or 50 mM LiCl for 24 h (*n*=3/group). Samples were resolved by capillary electrophoresis and stained with antibodies against phospho-Ser33/Ser37-β-catenin (A), total β-catenin (B), phospho-Ser9-GSK3β (C), total GSK3β (D), or GAPDH (E) to produce a chemiluminescent signal. The electropherogram peak area was quantified and expressed as mean±s.d., *P<0.05, two-tailed *t*-test.

Cell culture studies typically use lithium in the 10-30 mM range for GSK3 inhibition, because these concentrations produce inhibition without cytotoxicity (Cheng et al., 1983a,b; Hedgepeth et al., 1997; Hoeflich et al., 2000; Oreña et al., 2000; Kaidanovich and Eldar-Finkelman, 2002; Sadot et al., 2002; van Noort et al., 2002a,b; Zhang et al., 2003; Naito et al., 2004; Levina et al., 2004; Yang et al., 2006; Sievers et al., 2006; Chen et al., 2006; Valvezan et al., 2012). A dose-response analysis including and extending this range was conducted (Fig. 3; Figs S5, S6, and S7). At 50 mM, the highest concentration tested, cytotoxicity was evident. Lithium caused increased phospho-Ser9-GSK3β (Fig. 3C), confirming its efficacy as a GSK3 inhibitor that activates the positive feedback loop. In the conventional model of the canonical Wnt signaling pathway, GSK3 inhibition would cause β-catenin phosphorylation to decrease, and total protein levels of β-catenin to increase. However, neither of these effects were observed with lithium in A172 cells. Treatment with 10, 20, and 30 mM LiCl caused β-catenin phosphorylation to increase by 42%, 73%, and 104%, compared to untreated cells. In the same samples, the change in total β-catenin was +5%, –2%, and +5%, respectively. Li^+^ had no effect on β-catenin phosphorylation at concentrations of 5 mM and lower. In summary, lithium did appear to act as a GSK3 inhibitor in A172 cells, based on the phospho-Ser9-GSK3β result, but its effects on phospho- and total β-catenin were contrary to the usual expectations for a GSK3 inhibitor.

**Fig. 4.**
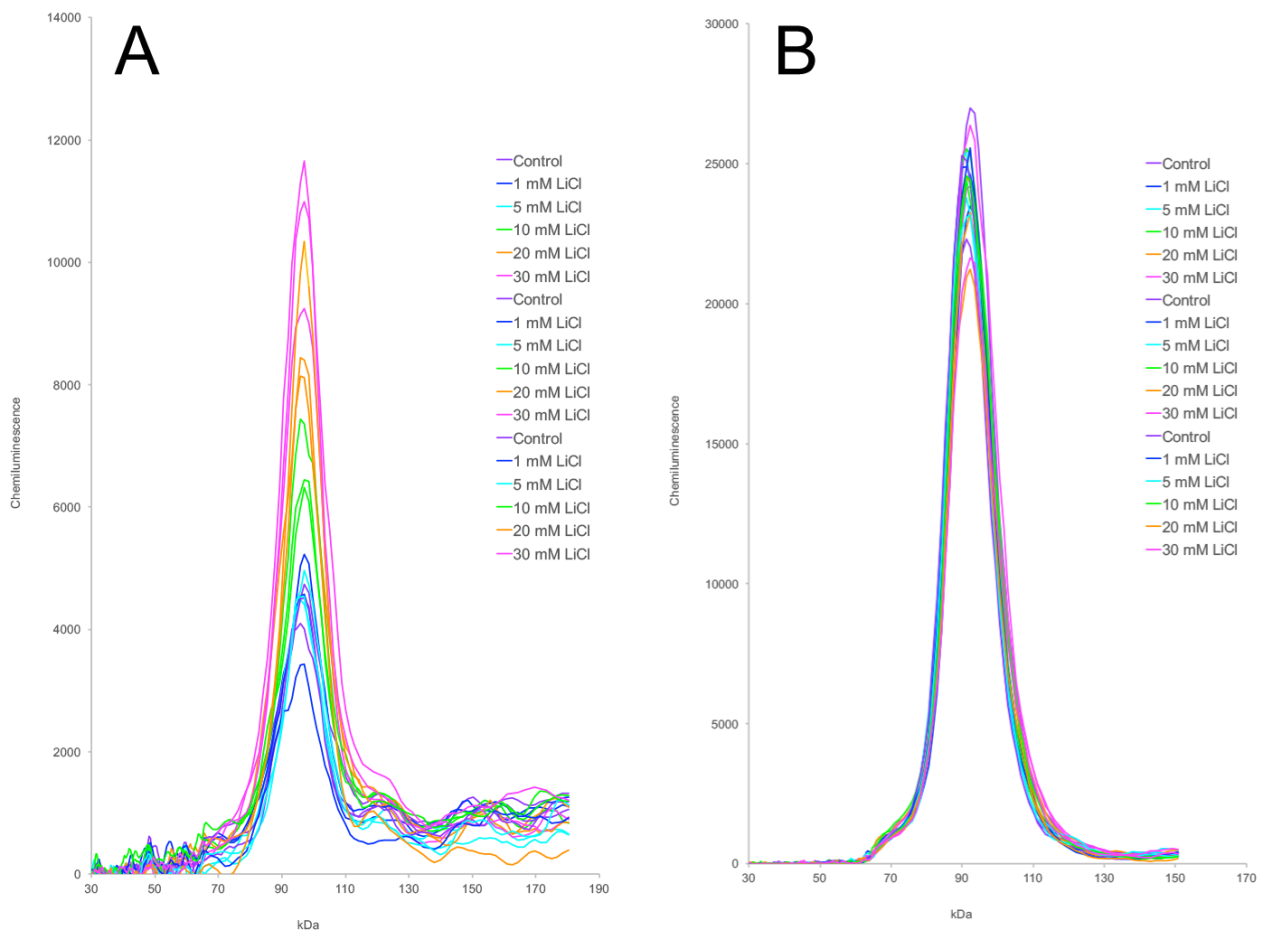
**Capillary electrophoresis electropherograms showing the effect of lithium on phosphorylation of β-catenin.** A172 cells were treated with 0 (purple), 1 (blue), 5 (cyan), 10 (green), 20 (orange), or 30 mM (pink) LiCl for 24 h (*n*=3/group). These are the same samples shown in Fig. 3 and Fig. S5, except that the 50 mM LiCl sample group is omitted to improve clarity. Samples were resolved by capillary electrophoresis and stained with antibodies against phospho-Ser33/Ser37-β-catenin (A) or total β-catenin (B). The electropherogram tracing for each of the 18 individual samples is shown.

The spatial distribution of chemiluminescence signal in each capillary can be depicted as a ‘virtual’ western blot. This is a convenient way to display a large set of results concisely (Figs. S5, S6, and S7). However, the electropherogram tracing is a more accurate representation of the raw data. The data are shown in this format for the most important subset of the lithium dose-response results, pertaining to phosphorylation of β-catenin (Fig. 4). The complete set of electropherogram peak areas is provided along with statistical analysis (Table S1). Depictions of normalized phospho-Ser33/Ser37-β-catenin and normalized phospho-Ser9-GSK3β [i.e. (phosphoprotein)/(corresponding total protein) ratios] are also provided (Fig. S8).

### Unlike Li^+^, Be^2+^ does not increase phosphorylation of β-catenin in A172 cells

In contrast to LiCl, BeSO_4_ at 10-1000 µM failed to stimulate phosphorylation of β-catenin at Ser33/Ser37 (Fig. S9). Instead, BeSO_4_ appeared to diminish phospho-Ser33/Ser37-β-catenin somewhat, especially at 100 µM, but a clear dose-dependent relationship was not evident. The phosphatase inhibitor calyculin A, which was used as a positive control, caused an increase in phospho-Ser33/Ser37-β-catenin, as expected (Fig. S9). BeSO_4_ had no appreciable effect on phosphorylation of GSK3β at Ser9 in A172 (Fig. S10), confirming previous observations for this cell type (Mudireddy et al., 2014). However, phospho-Ser9-GSK3β was clearly increased by calyculin A, the positive control (Fig. S10). Examination of GAPDH and total protein by capillary electrophoresis confirmed that the total protein levels in each sample were well-matched (Fig. S11). BeSO_4_ at 10-1000 µM did produce an increase in p21 protein expression (Fig. S12), which is a known response to Be^2+^ in this cell type (Gorjala and Gary, 2010; Gorjala et al., 2016), confirming the reliability of the BeSO_4_ treatments. The phosphatase inhibitor calyculin A had no effect on p21 expression (Fig. S12).

### Nuclear accumulation of β-catenin after lithium treatment

**Fig. 5.**
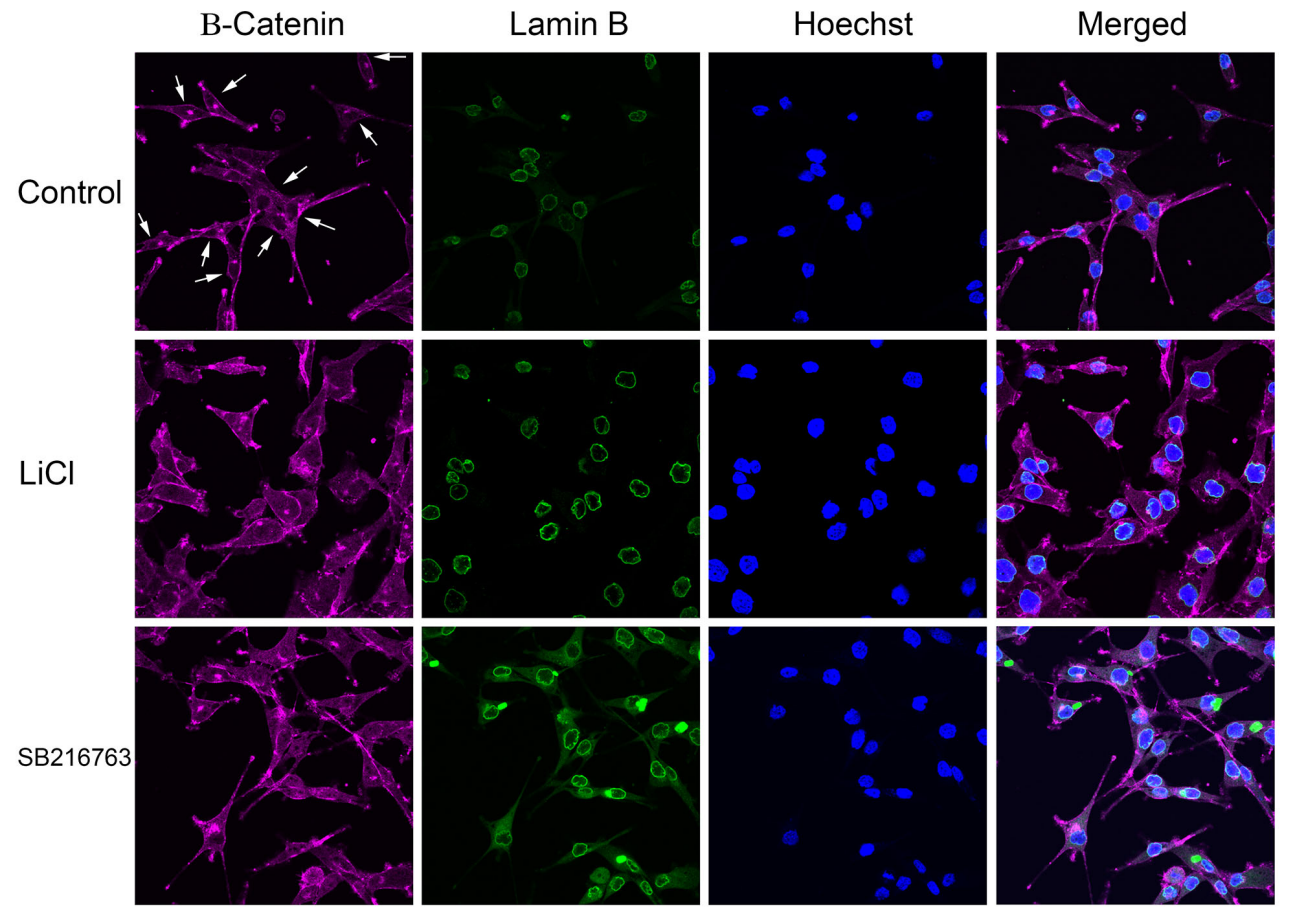
**Lithium induces nuclear accumulation of β-catenin in A172 cells.** Cells were untreated (top row), treated with 20 mM LiCl for 24 h (middle row), or treated with 20 µM SB216763 GSK3 inhibitor for 24 h (lower row), and analyzed by confocal microscopy. Arrows in the control image indicate nuclear regions that are devoid of staining (in some instances, the dark region is closely adjacent to one or more bright foci). The large green blobs in the lamin B channel are SB216763 aggregates; this compound has a known tendency to form aggregates that are intensely fluorescent at this excitation wavelength (Kirby et al., 2012).

In the conventional model of Wnt signaling, diminished GSK3 kinase activity causes β-catenin hypophosphorylation, enabling the protein to escape phosphorylation-directed degradation, which leads to the entry of β-catenin into the nucleus. Considering that lithium causes β-catenin hyperphosphorylation in A172 cells, we wondered whether β-catenin would enter the nucleus after lithium treatment in this cell type. To address this question, confocal microscopy was used to examine the subcellular distribution of β-catenin (Fig. 5). A172 cells were labeled with antibodies for β-catenin and lamin B, a component of the nuclear lamina that is located on the inner surface of the nuclear membrane. Hoechst 33342, a DNA-binding dye, was used to label chromatin. In untreated A172 cells, the absence of β-catenin staining in the nucleus created dark nuclear regions, indicated by arrows in Fig. 5, that contrast with the brighter cytoplasmic distribution. In lithium-treated cells, exclusion from the nucleus was reduced, and almost all of the cells showed a diffuse β-catenin staining throughout the nucleus whose intensity was similar to that of the cytoplasm. The increase in the staining intensity in the nuclear region due to accumulation of β-catenin demonstrated that this aspect of the lithium response remains consistent with the conventional expectations for GSK3 inhibition. The potent and selective cell-permeable GSK3 inhibitor SB216763 was used as a positive control treatment. SB216763 treatment causes an increase in nuclear β-catenin, and induces transcription of β-catenin-TCF/LEF regulated reporter genes (Coghlan et al., 2000; Piazza et al., 2010; Zhang et al., 2012). In A172 cells, SB216763 elicited a β-catenin nuclear translocation staining pattern comparable to lithium treatment.

### Li^+^ causes a decrease in axin

**Fig. 6.**
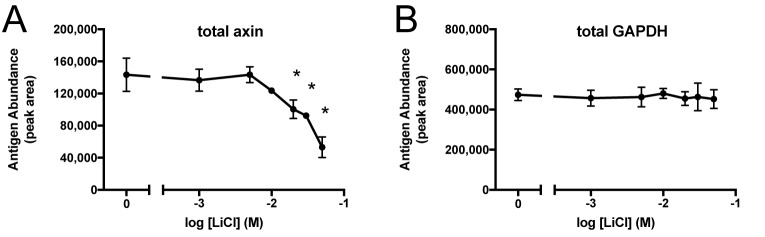
**Lithium causes dose-dependent depletion of axin in A172 cells.** Cells were treated with 0, 1, 5, 10, 20, 30, or 50 mM LiCl for 24 h (*n*=3/group). Samples were resolved by capillary electrophoresis and stained with antibodies against axin (A) or GAPDH (B). The electropherogram peak area was quantified and expressed as mean±s.d., *P<0.05, two-tailed *t*-test.

Although phosphorylation of β-catenin provides a signal for degradation in the conventional model of Wnt signaling, the phosphorylated form accumulated without degradation in lithium-treated A172 cells. One possible explanation for this observation could be that ubiquitination is somehow decoupled from phosphorylation in lithium-treated A172 cells. The scaffolding protein axin and the E3-ubiquitin ligase β-TrCP are each constituents of the β-catenin destruction complex (Stamos and Weis, 2013). If lithium were to interfere with the function of axin, that could have an effect on β-TrCP-mediated ubiquitination. Our preliminary western blot results showed that lithium treatment caused axin protein levels to decrease in A172 cells (data not shown). Therefore, a quantitative examination of the effect of lithium on axin was undertaken using the capillary electrophoresis method (Fig. 6). Lithium caused a dose-dependent depletion of axin in this cell type. After treatment with 10, 20, 30, or 50 mM LiCl, axin levels fell to 86%, 70%, 64%, and 37% of control, with the latter three results statistically significant. In contrast, the treatments had no effect on GAPDH, the housekeeping protein. The complete set of raw data and statistical analysis for the A172 axin experiment is provided (Table S1).

### The effects of Li^+^ on β-catenin phosphorylation and axin abundance are not unique to A172 cells

In order to gauge whether the unexpected effects of lithium treatment were specific to A172 cells or more broadly applicable, we chose four additional cell lines for analysis. Because A172 cells are from glioblastoma, we looked at two more human glioblastoma lines, U87MG and U251, to see whether glioma cells have a general tendency to respond to lithium in this manner. U87MG cells express wild-type p53 protein, whereas U251 cells express mutated p53. Both cell lines express β-catenin at moderate levels (Lee et al., 2013). To represent examples of non-glioma cell types, HT-1080 human fibrosarcoma and RKO human colon carcinoma cells were selected. These cell lines have been reported to express β-catenin at moderate levels (Sievers et al., 2006; Abdel-Rahman et al., 2016).

**Fig. 7.**
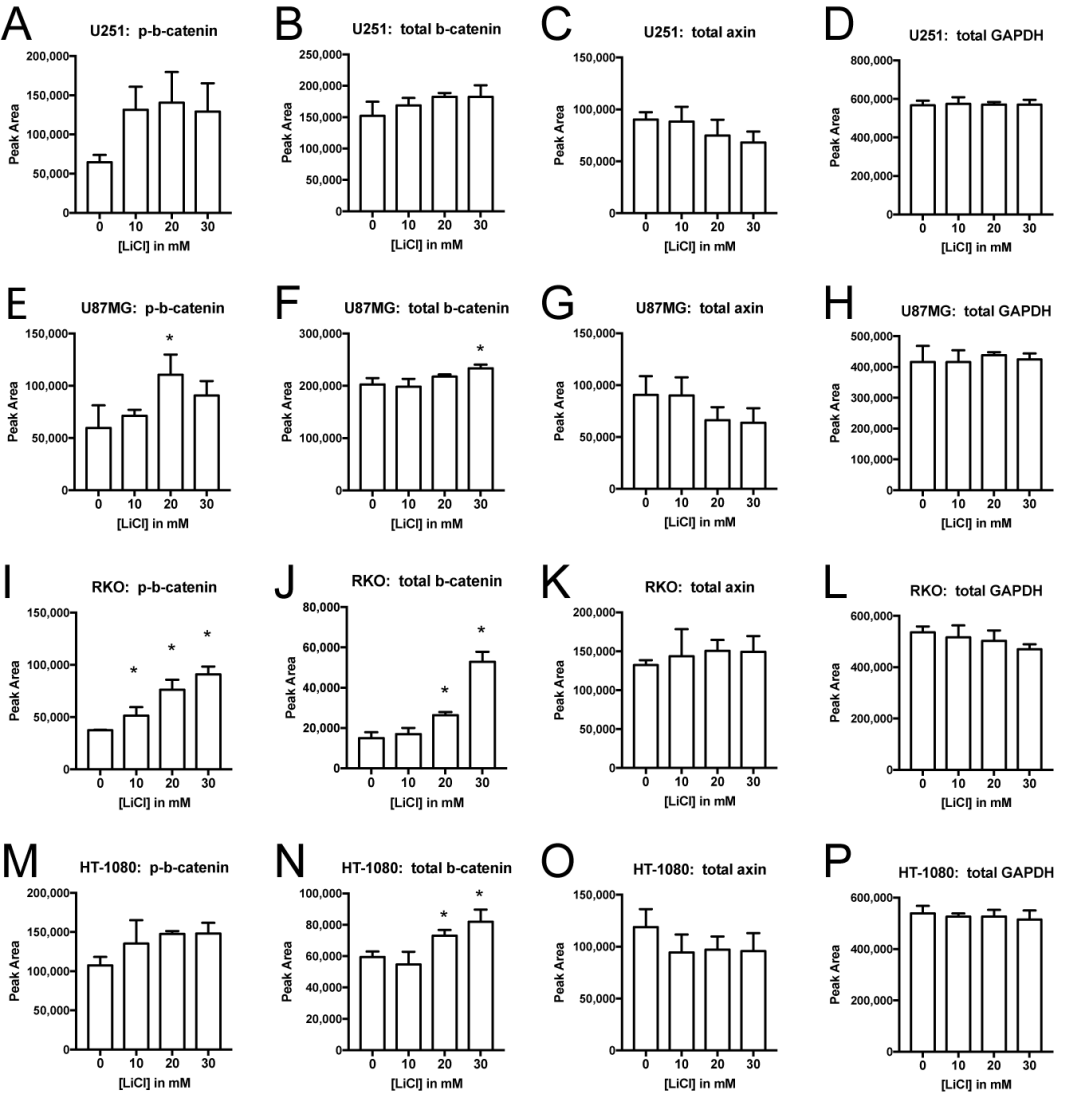
**Effects of lithium in other cell types.** U251 (A-D), U87MG (E-H), RKO (I-L), and HT-1080 (M-P) cells were treated with 0, 10, 20, or 30 mM LiCl for 24 h (*n*=3/group). Samples were resolved by capillary electrophoresis and stained with antibodies against phospho-Ser33/Ser37-β-catenin (A,E,I,M), total β-catenin (B,F,J,N), axin (C,G,K,O), or GAPDH (D,H,L,P). The electropherogram peak area was quantified and expressed as mean±s.d., *P<0.05, two-tailed *t*-test.

The two additional glioma cell lines responded to lithium in a manner that was generally similar to A172 cells, although the effects in A172 were more pronounced. Lithium caused a large increase in phospho-β-catenin in U251 and U87MG cells, and a proportionally much smaller increase in total β-catenin (Fig. 7). For U251 cells, 10, 20, and 30 mM LiCl caused phospho-β-catenin to increase by 104%, 118%, and 100%, whereas total β-catenin increased by only 11%, 20%, and 20% in these samples. Although the phospho-β-catenin increase was not statistically significant as defined by the criteria set out in the Materials and methods section, this was truly a borderline case. In pairwise comparisons with the control group, the lithium treatments yielded *t*-test *P*-values of 0.02, 0.03, and 0.04, respectively, but the ANOVA was just short of significance with *P*=0.056 (Table S1). The general trends in U87MG were fairly similar, but the magnitude of the effects was lower. Like A172, both U251 and U87MG displayed dose-dependent decreases in axin levels, down to 70% of control in the case of U87MG. The complete set of raw data and statistical analysis for the U251, U87MG, RKO, and HT-1080 experiments is provided (Table S1). Depictions of normalized phospho-Ser33/Ser37-β-catenin [i.e. (phospho-β-catenin)/(total β-catenin) ratios] are also provided (Fig. S13).

The RKO cell type provided an interesting contrast to the glioma cell lines. Lithium produced a massive increase in total β-catenin, raising it to 353% of control in the case of 30 mM LiCl, representing a 253% increase (Fig. 7). This is in line with the usual expectations for the effects of a GSK3 inhibitor in the canonical Wnt signaling pathway. Lithium did not diminish axin levels at all in RKO cells, providing another clear contrast with the glioma cells. An increase in phospho-β-catenin was observed in RKO cells, but unlike in the glioma cells, this increase was almost completely accounted for by the increase in total β-catenin. In RKO cells, the maximum increase in normalized phospho-β-catenin was 20% (at 10 mM), whereas the maximum increase was 96% (at 30 mM) for A172, 81% (at 10 mM) for U251, and 78% (at 20 mM) for U87MG (Figs S8 and S13, Table S1). With respect to all of the antigens, the responses of HT-1080 cells were intermediate between the contrasting responses of RKO cells and glioma cells.

## DISCUSSION

Lithium is a classic GSK3 inhibitor, and beryllium is a structurally related inhibitor. One of the most important GSK3 substrates is β-catenin. In A172 cells, Be^2+^ at 100 µM decreased phospho-β-catenin as seen in western blots (Fig. 1D) and in capillary electrophoresis (Fig. S9), but the magnitude of this effect relative to experimental variation was modest. In contrast, Li^+^ increased phospho-β-catenin (Figs 1, 2, 3 and 4). This was a surprising result, because lithium is regarded to be a GSK3 inhibitor, and GSK3 is the kinase responsible for phosphorylating β-catenin at positions Ser33, Ser37, and Thr41. Lithium had no effect at 1 and 5 mM, and it produced a concentration-dependent increase in phosphorylation at 10, 20, and 30 mM (Figs 3 and 4; Fig. S5). At 50 mM, a concentration that exhibits some cytotoxicity, phosphorylation began to decline yet still exceeded the levels seen in untreated cells.

Lithium-induced hyperphosphorylation of β-catenin was observed in two other glioma cell lines, showing that the response is not restricted to A172 cells, and suggesting that it may be common in this cell type. The phenomenon was also observed in the non-glial cell types RKO and HT-1080, but in these cell lines the change in phospho-β-catenin was more or less proportional to the change in total β-catenin. Even a proportional increase does not seem to comport with the standard view of canonical Wnt signaling. If the total pool of β-catenin increases because the subset of β-catenin that is phosphorylated is degraded, it is not clear why phospho-β-catenin should increase in proportion to total β-catenin, especially under conditions in which the kinase responsible for the phosphorylation is pharmacologically inhibited.

A172 cells possess wild-type p53. Lithium treatment activates p53 in A172 cells. There are known interactions between p53 signaling and the Wnt/GSK3/β-catenin signal transduction pathway, so experiments were designed to test whether p53 has a role in the unexpected hyperphosphorylation of β-catenin in response to lithium. However, this possibility was discounted after comparing normal A172 cells and a p53 knockdown A172 cell line. In A172 cells, the presence or absence of p53 function did not fundamentally alter the effects of lithium on β-catenin hyperphosphorylation. This conclusion was further reinforced by comparing U87MG (wild-type p53) and U251 (mutated p53), which gave similar results.

Studies with various cell types have shown that lithium treatment induces accumulation of total β-catenin protein (similar to the RKO result in Fig. 7), and it is usually inferred that the mechanism involves avoidance of degradation due to β-catenin hypophosphorylation as a result of GSK3 inhibition. Direct examination of the phosphorylation status of β-catenin after lithium treatment is less commonly reported, but nonetheless documentation of hypophosphorylation in response to lithium is plentiful (van Noort et al., 2002a,b; Sadot et al., 2002; Sinha et al., 2005; Yang et al., 2006; Takatani et al., 2011; Zhu et al., 2011; Tian et al., 2014). As a GSK3 kinase inhibitor, lithium should generally decrease Ser/Thr phosphorylation events in treated cells. In this paper, two examples contrary to this proposition are shown: (1) lithium-induced hyperphosphorylation at Ser9-GSK3β; and (2) lithium-induced hyperphosphorylation at the Ser33 cluster of β-catenin. The first hyperphosphorylation event is well-known in the literature and has been characterized mechanistically, whereas the second hyperphosphorylation is more puzzling. There are important distinctions between the two cases. PKB(Akt) is the kinase predominantly responsible for phosphorylating Ser9-GSK3β (Cross et al., 1995), whereas GSK3β is the kinase that phosphorylates Ser33 of β-catenin. Lithium has no direct effect on the Ser9 kinase (PKB), but it directly inhibits the Ser33 kinase (GSK3β), making hyperphosphorylation at Ser33 seem more implausible. Steady-state phosphorylation levels are always a reflection of the balance between kinase and phosphatase activities. It has been shown that GSK3 activity is necessary to maintain the activity of PP1, the phosphatase responsible for hydrolyzing the Ser9 modification. GSK3β phosphorylates I-2, an inhibitory subunit of PP1, which relieves the inhibitory effect of I-2, thereby promoting PP1 phosphatase activity (Zhang et al., 2003). Thus, lithium treatment reduces GSK3 activity, which in turn reduces PP1 activity, which alters the kinase/phosphatase balance with respect to the Ser9 position (i.e. PKB versus PP1), which results in hyperphosphorylation at Ser9. Regarding the more novel Ser33 observation, lithium may act, either directly or indirectly, to inhibit a phosphatase activity that is responsible for phosphate hydrolysis at the Ser33 cluster of β-catenin. The PP2A phosphatase is a potential candidate for such activity, as it has been shown to be important in dephosphorylating Ser33 (Su et al., 2008; Zhang et al., 2009). The PP2A catalytic subunit associates with a variety of regulatory subunits that determine substrate specificity, and some form of PP2A could be involved in mediating the effects of lithium in A172 cells. *In vitro* phosphatase assays show that lithium does not inhibit PP2A directly (Chen et al., 2006), but it has been suggested that lithium treatment of cells could lead to PP2A inhibition indirectly (Mora et al., 2002; Chen et al., 2006).

Although lithium treatment promotes hypophosphorylation of β-catenin in many cell types (van Noort et al., 2002a,b; Sadot et al., 2002; Sinha et al., 2005; Yang et al., 2006; Takatani et al., 2011; Zhu et al., 2011; Tian et al., 2014), it promotes hyperphosphorylation in A172, U251, and U87MG cells, as shown here. It may be that the kinase/phosphatase balance in glioma cells is unusually well-suited to revealing the existence of a GSK3-dependent phosphatase activity that acts on the Ser33 cluster of β-catenin. This explanation, if correct, would indicate a new type of kinase/phosphatase feedback relationship, analogous to the more familiar regulation of PP1. Primary human T cells are another cell type in which a paradoxical relationship exists between GSK3 activity and β-catenin phosphorylation. T cell receptor stimulation causes GSK3 activity to decrease (inferred from changes in p-Ser9-GSK3β) and yet p-β-catenin levels increase (Lovatt and Bijlmakers, 2010).

If phospho-β-catenin were rapidly degraded, that would tend to prevent the accumulation of this phosphoprotein. However, this does not appear to be the case in lithium-treated A172 cells. Proteasome-mediated turnover of phospho-β-catenin does occur in A172 cells, as shown by the effects of MG-132 on untreated control cells (Fig. 1B,E). However, in lithium-treated cells, the addition of MG-132 does not appear to raise levels of the phospho-protein very much (Fig. 1B,E), raising the possibility that ubiquitination of phospho-β-catenin could somehow be impaired by lithium treatment. There is some precedent for the dissociation of β-catenin phosphorylation and ubiquitination. For example, c-FLIP-L, which is expressed in a variety of cancers, inhibits the ubiquitination and degradation of β-catenin at a step downstream of GSK3-mediated phosphorylation (Naito et al., 2004). Our results suggest that lithium-induced axin depletion could be responsible for decoupling β-catenin phosphorylation from subsequent ubiquitination and degradation. The β-catenin destruction complex contains both axin and the E3-ubiquitin ligase β-TrCP, which targets phospho-β-catenin. Stoichiometrically, the cellular concentration of axin is considered to be a limiting factor in the formation of the destruction complex, so the composition and functioning of this macromolecular system is sensitive to axin levels. It may be that depletion of axin by lithium enables phospho-β-catenin to evade β-TrCP and accumulate, at least in glioma cells. Recently, the axin/WDR26 complex has been shown to function in the ubiquitination and degradation of β-catenin (Goto et al., 2016), providing further support for this hypothesis. Correlations consistent with this idea were observed. A172 cells exhibited the greatest lithium-induced decline in axin and the largest lithium-induced increase in phospho-β-catenin. U251 and U87MG cells had lesser declines in axin and more modest increases in the phosphoprotein. The situation seems to be different in RKO cells. Basal levels of β-catenin appeared to be low in this cell type compared to the others tested, presumably due to robust basal degradation rates in accordance with the conventional view of protein turnover in the Wnt signaling model. Lithium caused β-catenin levels to soar in RKO cells, but without interfering with axin levels. Phospho-β-catenin levels were surprisingly high in lithium-treated RKO cells, an observation not well explained by the standard model. This study may motivate investigators to examine β-catenin phosphorylation status in other systems where treatment-induced β-catenin accumulation is observed.

The surprising ability of A172 and other glioblastoma cells to accumulate phospho-β-catenin in response to lithium treatment may have some parallels with the behavior of HEK293T human embryonic kidney cells. Lithium is generally regarded to mimic Wnt stimulation. When the Wnt signaling pathway of HEK293T cells is stimulated by treatment with the Wnt3a ligand, accumulation of β-catenin phosphorylated at Ser33/Ser 37/Thr41 is observed (Li et al., 2012; Gerlach et al., 2014). In the conventional model, phospho-β-catenin would be targeted for rapid degradation, but the authors proposed that Wnt-stimulation in HEK293T cells creates a condition in which ubiquitination and degradation of phospho-β-catenin is curtailed. Thus, with respect to accumulation of phospho-β-catenin, lithium treatment of glioma cells resembles Wnt stimulation of HEK293T cells. There are further parallels between the two systems. Wnt signaling in HEK293T cells causes depletion of axin protein (Li et al., 2012; Gerlach et al., 2014). We observed that lithium treatment caused axin to decrease in all three of the glioma lines studied. In each of these systems, loss of axin is an attractive candidate to explain how β-catenin phosphorylation and ubiquitination/degradation could be decoupled by Wnt stimulation or its analog, lithium treatment.

As a therapeutic agent, lithium is currently administered for bipolar disorder, and its use may someday be expanded due to its potential in Alzheimer's disease. It is important to understand the actions of lithium on signaling pathways, particularly in brain tissue, in order to make reasonable hypotheses regarding therapeutic mechanism of action, as well as to become alert to the types of side effects that might arise from lithium therapy. This study shows that lithium does not necessarily affect the phosphorylation status of GSK3 substrates in the manner that would be predicted by standard models of the kinase network.

## MATERIALS AND METHODS

### Cell culture

NIH-3T3 mouse embryo fibroblasts, A172 human glioblastoma, U251 human glioblastoma, U87MG human glioblastoma, RKO human colon carcinoma, and HT-1080 human fibrosarcoma cells were obtained from American Type Culture Collection (Manassas, VA, USA). The generation of stable p53 knockdown A172 cell lines using lentivirus has been previously described (Gorjala et al., 2016). These cells express shRNA that knocks down p53 expression, or a non-targeted shRNA generated in parallel that is used as a control. All cells were grown in RPMI 1640 supplemented with 10% fetal bovine serum (10% calf serum for NIH-3T3 cells), 25 mM HEPES and 1× antibiotic-antimycotic (Invitrogen-Gibco) at 37°C in 5% CO_2_.

### Chemicals

Stocks of BeSO_4_•4H_2_O (Fluka division of Sigma-Aldrich, St. Louis, MO, USA) and LiCl (Sigma) were prepared in ultrapure water and sterile filtered. MG-132 and SB216763 were from Santa Cruz Biotechnology (Santa Cruz, CA, USA) and calyculin A was from Cell Signaling Technology (Danvers, MA, USA). Hoechst 33342 dye was from Sigma.

### Western blotting

Cells were grown in 100 mm culture dishes and harvested by trypsinization. Cells were washed twice with phosphate-buffered saline and total cell lysates were prepared using M-PER lysis reagent (Pierce #78501) supplemented with protease (Halt protease inhibitor cocktail, Pierce #78410) and phosphatase inhibitors (20 mM sodium fluoride, 10 mM beta-glycerophosphate, 0.1 mM sodium ortho vanadate, 20 mM *p*-nitrophenyl phosphate). Lysate was clarified by centrifugation. A portion of the supernatant was mixed with Laemmli SDS sample buffer and boiled immediately, and a portion was used in a BCA assay (Pierce #23227) to measure total protein concentration. Normalized protein samples were resolved by SDS-PAGE, transferred to PVDF membrane (Millipore #IPFL20200 or Bio-Rad #162-0255), and blocked with 10% milk or, when probing with phospho-antibodies, StartingBlock™T20-TBS (Thermo Scientific Pierce #37543). Primary antibody-labeled blots were incubated with their respective HRP-conjugated secondary antibodies and developed with ECL-Plus (GE Healthcare Life Sciences) or Clarity Western ECL substrate (Bio-Rad #170-5061). The following primary antibodies were used for western blots: β-catenin mouse monoclonal (clone E5, #sc-7963, Santa Cruz Biotechnology), phospho-Ser33-β-catenin rabbit polyclonal (#sc-16743-R, Santa Cruz Biotechnology), p53 mouse monoclonal (clone DO-1, # sc-126, Santa Cruz Biotechnology), actin goat polyclonal (#sc-1615 Santa Cruz Biotechnology).

### Capillary electrophoresis

Cells were treated as indicated and harvested by trypsinization, then washed with PBS. In the ‘Development of a capillary electrophoresis method’ experiment, cells were lysed in 0.1× SDS-containing Sample Buffer (ProteinSimple, San Jose, CA, USA) supplemented with protease and phosphatase inhibitors, and heated at 95°C for 3 min. For all other experiments, the cells were lysed in M-PER lysis reagent supplemented with protease and phosphatase inhibitors. The protein concentration of lysates was measured using the BCA assay, and the same amount of protein was loaded into each capillary for a particular antigen analysis series. The total protein amount per 5 µl is listed for each antigen (Table S1), however the instrument draws only a portion of this for analysis. Lysates were mixed with SDS-containing Sample Buffer to give a 1× final concentration, fluorescent internal control size markers (1 kD, 29 kD, 230 kD) were included (ProteinSimple, San Jose, CA, USA), and the sample mixture was heated to 95°C for 5 min. Samples were run on 12-230 kD size assay capillaries in a Wes capillary electrophoresis system (ProteinSimple). For each series, one capillary containing a biotinylated size ladder (12, 40, 66, 116, 180, and 230 kD) was always included in the run. Primary antibody concentrations (described below) were optimized in pilot experiments. HRP-conjugated mouse and rabbit secondary antibodies, and chemiluminescence detection reagents, were from ProteinSimple. Capillary electrophoresis data analysis, including peak area determinations, was done using Compass software (ProteinSimple). Total protein labeling reagent was from ProteinSimple. The following primary antibodies were used for capillary electrophoresis, at the indicated dilutions: 1:500 GAPDH rabbit polyclonal (#sc-25778, Santa Cruz Biotechnology), 1:100 GSK3β rabbit polyclonal (#sc-9166, Santa Cruz Biotechnology), 1:50 phospho-Ser9-GSK3β rabbit polyclonal (#9336, Cell Signaling Technology), 1:250 β-catenin rabbit monoclonal (clone D10A8, #8480, Cell Signaling Technology), 1:50 phospho-Ser33/Ser37-β-catenin rabbit polyclonal (#2009, Cell Signaling Technology), 1:50 phospho-Ser33/Ser37/Thr41-β-catenin rabbit polyclonal (#9561, Cell Signaling Technology), 1:10 p21 mouse monoclonal (clone F-5, #sc-6246, Santa Cruz Biotechnology), 1:100 axin1 rabbit monoclonal (#3323, Cell Signaling Technology).

### Statistical analysis

Results are plotted as mean±standard deviation. Experiments with multiple treatment groups were first analyzed by one-way ANOVA, with *P*<0.05 as the criterion for overall significance. If significance was observed, two-tailed unpaired *t*-tests were used for individual comparisons between a specific drug concentration treatment group and the untreated control group, with *P*<0.05 considered significant. For statistics on ratios (i.e. the [phosphoprotein]/[total protein] normalization), the log[total protein] was subtracted from the log[phosphoprotein] to obtain a log[difference]. This data transformation is preferable because, unlike ratios, arithmetic combinations of logs exhibit a Gaussian distribution (Keene, 1995). The standard deviation calculations and significance tests were conducted on the log[difference] values, then the anti-logs were taken to back-transform the data for graphical display. Statistical analysis was conducted using Microsoft Excel for Mac, 2016 version.

### Confocal microscopy

A172 cells were grown in MatTek glass-bottom dishes (with No. 1.5 coverslip), treated as indicated, washed with phosphate-buffered saline (PBS) supplemented with 1 mM CaCl_2_ and 0.5 mM MgCl_2_, then fixed using 4% paraformaldehyde in PBS. The cells were permeabilized with 0.2% Tween20 in PBS with Ca^2+^ and Mg^2+^, then blocked in antibody incubation buffer (1% bovine serum albumin in 0.2% Tween20 in PBS with Ca^2+^ and Mg^2+^). Cells were double-labeled overnight at 4°C with mouse monoclonal anti-β-catenin antibody (#sc-7963, Santa Cruz Biotechnology) and goat polyclonal anti-lamin B antibody (#sc-6216, Santa Cruz Biotechnology). Secondary antibodies were Alexa Fluor 647-conjugated anti-mouse IgG (#4410, Cell Signaling Technology), and FITC-conjugated anti-goat IgG (#sc-2024, Santa Cruz Biotechnology). The DNA dye Hoechst 33342 was used at 1 µg/ml to stain chromatin. Images were collected using a Nikon A1R confocal laser scanning microscopy system.

## Supplementary Material

Supplementary information
