# Supplementary material for: The GSK3 kinase inhibitor lithium produces unexpected hyperphosphorylation of β-catenin, a GSK3 substrate, in human glioblastoma cells
Source: Biol Open. 2017 Dec 6;7(1):bio030874. doi: 10.1242/bio.030874 (PMC5829510; doi:10.1242/bio.030874)
Supplement: Supplementary information [file biolopen-7-030874-s1.pdf]

© 2018. Published by The Company of Biologists Ltd | Biology Open (2018): doi:10.1242/bio.030874: Supplementary information

FIRST PERSON

First person – Ata ur Rahman Mohammed Abdul

First Person is a series of interviews with the first authors of a selection of papers published in Biology Open, helping early-career researchers promote themselves alongside their papers. Ata ur Rahman Mohammed Abdul is first author on 'The GSK3 kinase inhibitor lithium produces unexpected hyperphosphorylation of β-catenin, a GSK3 substrate, in human glioblastoma cells', published in BiO. Ata is a Quality Control Associate in the lab of Ronald K. Gary at Chromak Research Inc., USA, investigating the effects of small molecule inhibitors such as lithium and beryllium on the cellular enzyme glycogen synthase kinase 3 beta (GSK3β).

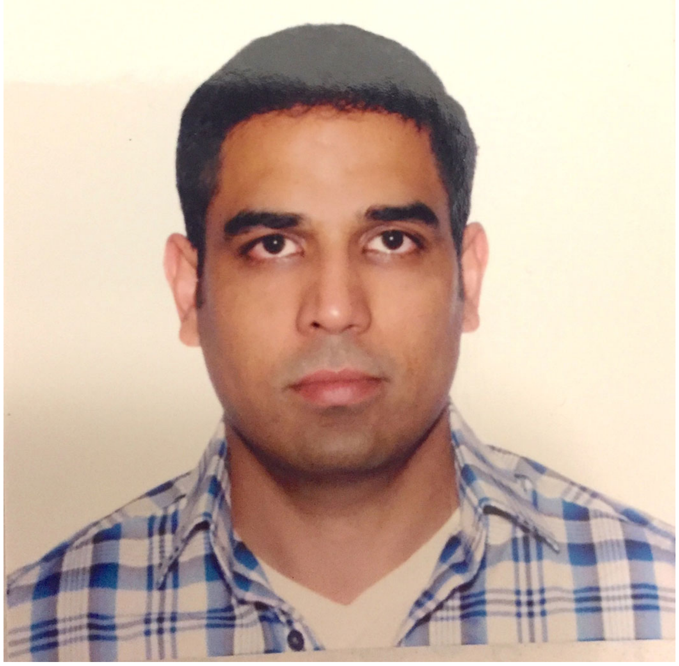

Ata ur Rahman Mohammed Abdul

What is your scientific background and the general focus of your lab?

I completed my PhD in the Department of Chemistry and Biochemistry at University of Nevada, Las Vegas, under the guidance of Dr Ronald K. Gary. As a graduate student I used small molecule inhibitors such as lithium and beryllium to study their effects on an important cellular enzyme known as glycogen synthase kinase 3 beta (GSK3β). Our work established that beryllium is an extremely potent GSK3β inhibitor compared to the other well-established small molecule inhibitor GSK3β inhibitor, lithium. Part of our research also documented the biological effects of beryllium on different biological pathways, which is a first. I currently work in the area of quality control and bio-analytics.

How would you explain the main findings of your paper to non-scientific family and friends?

Lithium is a widely used GSK3β inhibitor and is used as a therapeutic agent for treating bipolar disorder. One of the most striking observations of the lithium-induced GSK3β inhibitor is the decrease in phosphorylation levels of an important GSK3β substrate known as β-catenin. However, in our studies it was observed consistently that lithium caused an increase in the phosphorylation levels of β-catenin. It is a unique observation because it is quite opposite to the expected results. These results indicate that there are some aspects of lithium-GSK3β interactions that we don't understand well yet.

> **"The main theme of our publication i.e. 'the lithium-induced hyperphosphorylation of GSK3' was a totally unexpected result."**

What are the potential implications of these results for your field of research?

β-catenin is an important substrate of the GSK3β enzyme. GSK3β phosphorylates β-catenin and marks it for proteasomal degradation. The inhibition of GSK3β by lithium is expected to decrease phospho-β-catenin levels and ultimately stabilize the total β-catenin levels. β-catenin is a transcription factor that regulates the expression of certain oncogenes, hence its stabilization can have potential oncogenic effects. If lithium is used as a therapeutic agent it always carries the risk of inducing oncogenic genes such as phospho-β-catenin instead of decreasing them. This study indicates that other players apart from GSK3β are involved in regulating phospho-β-catenin levels and warrant investigation further.

What has surprised you the most while conducting your research?

The main theme of our publication i.e. 'the lithium-induced hyperphosphorylation of GSK3' was a totally unexpected result, observed in an experiment planned to test a totally different hypothesis. Initially, the results were ignored; however, the consistent reproducibility of similar observations in different cell lines backed by alternate experimental data validated this unexpected result.

What, in your opinion, are some of the greatest achievements in your field and how has this influenced your research?

A thorough literature survey can provide a young scientist with a sound perspective to plan experiments and execute them. A researcher should not be scared of planning a bold experiment or trying out a new hypothesis. Never ignore any observation especially if it is consistently reproduced. Apart from practicing good research it is extremely important to invest time in networking and collaborating to grow professionally.

What's next for you?

Next I would like to work in the area of cell therapy and regenerative medicine. I would love to work on CAR-T therapy and make a very

Ata ur Rahman Mohammed Abdul's contact details: Chromak Research Inc., Somerset, USA
E-mail: ata.abdul@paradisevalley.edu

 Biology Open (2018): doi:10.1242/bio.030874: Supplementary information

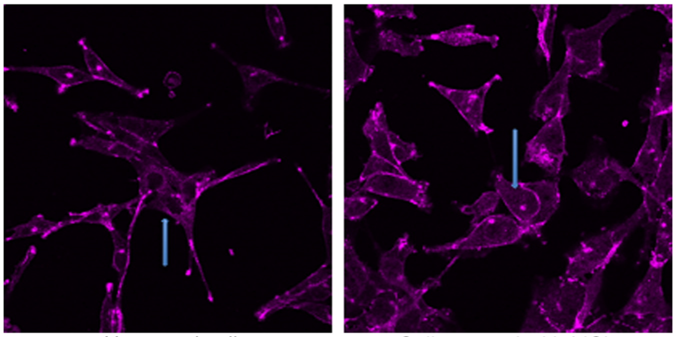

Untreated cells Cells treated with LiCl

Lithium treatment leads to accumulation of β-catenin in the nucleus (indicated by arrow).

small contribution that could make a huge difference to someone's life.

Reference

Abdul, A. R. M., De Silva, B. and Gary, R. K. (2018). The GSK3 kinase inhibitor lithium produces unexpected hyperphosphorylation of β-catenin, a GSK3 substrate, in human glioblastoma cells. *Biol. Open* **7**, doi:10.1242/bio.030874.

Biology Open
